# Using zebrafish to understand reciprocal interactions between the nervous and immune systems and the microbial world

**DOI:** 10.1186/s12974-022-02506-x

**Published:** 2022-06-28

**Authors:** Jean-Pierre Levraud, John F. Rawls, Anne E. Clatworthy

**Affiliations:** 1 Université Paris-Saclay, CNRS, Institut Pasteur, Université Paris-Cité, Institut des Neurosciences Paris-Saclay, 91400 Saclay, France; 2 Department of Molecular Genetics and Microbiology, Duke Microbiome Center, Duke University School of Medicine, 213 Research Drive, Durham, NC 27710 USA; 3 Broad Institute of MIT and Harvard, 415 Main Street, Cambridge, MA 02142 USA; 4 Department of Molecular Biology and Center for Computational and Integrative Biology, Massachusetts General Hospital, 185 Cambridge Street, Boston, MA 02114 USA

**Keywords:** Zebrafish, Neuro-immune interactions, Microbiota, Homeostasis

## Abstract

Animals rely heavily on their nervous and immune systems to perceive and survive within their environment. Despite the traditional view of the brain as an immunologically privileged organ, these two systems interact with major consequences. Furthermore, microorganisms within their environment are major sources of stimuli and can establish relationships with animal hosts that range from pathogenic to mutualistic. Research from a variety of human and experimental animal systems are revealing that reciprocal interactions between microbiota and the nervous and immune systems contribute significantly to normal development, homeostasis, and disease. The zebrafish has emerged as an outstanding model within which to interrogate these interactions due to facile genetic and microbial manipulation and optical transparency facilitating in vivo imaging. This review summarizes recent studies that have used the zebrafish for analysis of bidirectional control between the immune and nervous systems, the nervous system and the microbiota, and the microbiota and immune system in zebrafish during development that promotes homeostasis between these systems. We also describe how the zebrafish have contributed to our understanding of the interconnections between these systems during infection in fish and how perturbations may result in pathology.

## Background

It is increasingly appreciated that deep, reciprocal interconnections between the immune system, the nervous system and the microbiota exist in vertebrates and are important for the development and homeostasis of each system as well as the health of the organism as a whole. For example, interactions between the immune and nervous systems establish normal neural connectivity in the brain and immune cell numbers in the periphery, while the enteric nervous system plays a major role in shaping gut microbial ecology, which in turn can influence behavior patterns. The immune system itself is important for control of microbiota composition, which is known to be a major determinant of susceptibility to intestinal pathogens. Thus, it is clear that these systems do interact to promote normal development and homeostasis. In addition, coordination between these systems is important for a return to homeostasis when an organism is faced with an external threat such as infection or injury. Finally, dysregulated interactions between these systems can result in pathology (*e.g.*, meningitis).

As a model organism, zebrafish is perhaps uniquely positioned to shed insight into interactions between the immune system, the nervous system, and the microbiota. The optical transparency of zebrafish embryos and larvae coupled with the increasing breadth of transgenic fish lines where cells of interest are fluorescently labeled facilitate the visualization of immune, neural, and microbiota interactions in an intact living vertebrate in real time during development and in response to infection or injury. Moreover, zebrafish are amenable to both classical and chemical genetics, and thus perturbing various genes of interest in order to investigate their contribution to immune, nervous system and microbiota interactions is relatively facile. Finally, the zebrafish immune system is very similar to mammals and includes both adaptive and innate immune cells (B cells, T cells, macrophages, neutrophils, etc.) and soluble immune mediators such as cytokines and complement proteins [1]. Strong homologies between brain structures, cells and genetic programs are also notable between zebrafish and mammals [2]. Given these features, zebrafish are a powerful model to explore the depth and complexity of the interactions between the nervous system, the immune system and the microbiota that are important to upholding organismal health in a living vertebrate.

Here, we review the available literature for studies of bidirectional control between the immune and nervous systems, the nervous system and the microbiota, and the microbiota and immune system in zebrafish during development that promotes homeostasis between these systems (part II). We then explore the interconnection of these systems during infection in fish (part III) and how perturbations may result in pathology (part IV).

## Homeostatic control between the immune system, the nervous system and the microbiota

### Immune control of the nervous system

**Fig. 1:**
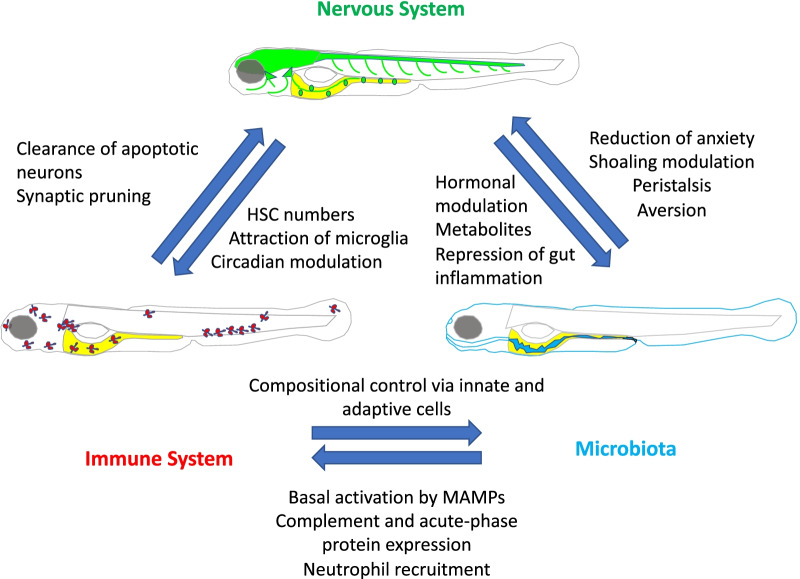
Examples of homeostatic reciprocal interactions between the nervous system, immune system, and microbiota, elucidated in the zebrafish model

In vertebrates there is clear evidence of immune control of normal nervous system development from cellular to behavioral levels. At the cellular level, it has long been recognized that neuronal apoptosis is a critical mechanism promoting normal neural connectivity in the developing brain. This process certainly occurs in zebrafish as early as 48 hours post-fertilization (hpf), when dead and dying neurons are observed and microglial precursors, cells of hematopoietic origin that are destined to differentiate into resident macrophages in the central nervous system (CNS), begin trafficking to the brain to clear apoptotic bodies [3]. While microglial clearance of apoptotic bodies in the brain is a clear example of homeostatic immune control of nervous system development, this control is bidirectional (Fig. 1) as is evidenced by recent work showing that it is neuronal apoptosis itself that recruits microglial precursors into the brain, in part via nucleotide signaling, resulting in the normal expansion and distribution of microglia throughout the brain [4].

At the behavioral level, there is some evidence of immune control of social behaviors, as mice deficient in lymphocytes have social deficits and display hyperconnectivity in fronto-cortical brain regions. These defects were shown to be driven by a lack of IFN-γ release from meningeal T cells that in wild-type animals contributes to tonic GABAergic signaling in the brain. While this study was primarily conducted in mice, the authors show that in addition to rodents, zebrafish and fruit flies upregulate IFN-γ and JAK–STAT-inducible genes in a social context. This work suggested that IFN-γ signaling and the ability to mount a robust pathogen defense is co-evolutionarily linked in animals that aggregate more closely with other individuals in a group, where pathogen spread is more likely [5].

While there is certainly immune control of normal neural connectivity, the immune system also plays a pivotal role in the regeneration of tissue following injury. Unlike mammals, zebrafish can regenerate neural cells and thus are a valuable model in understanding how neural regeneration can occur in the hope of translating such knowledge into future therapeutics to treat traumatic brain and spinal cord injuries and neurodegenerative disorders. In adult zebrafish following brain injury, leukocytes traffic to sites of injury where pro-inflammatory cytokines (TNFα, IL1β, and IL-8) are expressed and it is the inflammation itself that enhances proliferation of neuroprogenitor cells [6]. When adaptive immunity is present in adult fish, there is a requirement for regulatory T cells (Tregs) in spinal cord regeneration. Tregs home to sites of tissue damage, such as damaged spinal cord tissue, and release regenerative factors specific for the damaged organ (e.g., neurotrophin 3 in damaged spinal cord tissue) and it is the regenerative factor rather than anti-inflammatory cytokines (e.g., IL-10) expressed by Tregs that promotes proliferation of neural progenitor cells [7]. In a final example of immune control of neural regeneration in a model of Alzheimer’s disease, IL-4 produced by microglia is sufficient to induce the proliferation of neuroprogenitor cells through the IL-4R/STAT6 pathway in areas of adult zebrafish brains suffering neurodegeneration as a result of Amyloid β42 toxicity [8]**.**

### Neural control of the immune system

#### Hypothalamus–pituitary–adrenal axis control of immune cell numbers

Similar to apoptotic neuronal recruitment of microglial precursors to the developing brain described above, another example of neural control of the immune system is the regulation of hematopoietic stem cell (HSC) numbers in the periphery by the central nervous system (CNS; Fig. 1). In developing zebrafish embryos, stressors such as hypoxia have been shown to stimulate serotonin production specifically in the CNS. Serotonergic stimulation of the hypothalamic–pituitary–adrenal/interrenal (HPA/I) stress response axis then acts via the glucocorticoid receptor to increase HSC numbers in the periphery ultimately increasing the numbers of available immune cells [9]. However, just as in mammals, dexamethasone treatment of zebrafish larvae has the short-term effect of depleting thymocytes [10].

#### Circadian rhythms and immunity

In mice, HSC release is also regulated daily by circadian oscillations with two daily peaks of HSCs released at the onset of light and darkness [11]**.** While it is unclear whether circadian oscillations regulate the release of HSCs in zebrafish, circadian rhythms do have a large impact on the immune response in zebrafish and can be considered another example of neural control of the immune system.

In mammals, neuro-endocrine interactions between the suprachiasmatic nuclei (SCN) in the hypothalamus and the HPA axis synchronize circadian rhythm control of immune responses in peripheral tissues [12]. Circadian rhythms are endogenous oscillations that tune an organism’s physiological processes to a ~ 24-h light–dark cycle. These oscillations exert temporal control over a wide range of behavioral activities and cellular and organ system functions including immune functions such as daily egress of innate immune cells from the bone marrow to the blood and immune organs, neutrophil trafficking, localization and turnover, macrophage phagocytic activity and cytokine release [13]. At the cellular level, circadian oscillations are maintained by sets of conserved transcription factors that execute an interlocked, rhythmic, self-perpetuating transcription- translation feedback loop. These transcription factors constitute the molecular clock machinery, are expressed in all major organs and tissues of the body and either directly or indirectly exert control over the range of processes described above.

In mammals, peripheral clocks are synchronized to the environmental light–dark cycle by the master or central clock located in the suprachiasmatic nuclei (SCN) in the hypothalamus where light input from the environment is transmitted from the lens of the eye through the retinohypothalamic tract to ~ 20,000 neurons in the SCN [12]. Neuro-endocrine interactions between the SCN and the HPA axis ultimately impose synchrony on peripheral clocks in tissues throughout the organism. However, this hierarchical structure is not common to all vertebrates. In zebrafish, there is no SCN structure [14]. Further, molecular clocks in all cells and organs in zebrafish can be directly entrained by light [15], which has driven the question of whether there is a need for a ‘master’ clock in fish. Recent work has suggested that fish may coordinate circadian oscillations throughout the organism using several clock centers through a ‘multiple pacemaker system,’ similar to birds [16]. At least one of these clock centers is located in the pineal gland, where intrinsic circadian oscillations are directly entrained by light through cells with retinal cone photo-receptor-like characteristics, which then drive melatonin production [17]. While coordination of circadian oscillations is more decentralized in fish than in mammals, this does not necessarily imply that neuro-endocrine interactions are not important for modulation of these oscillations between tissues in fish and ultimately circadian control of immune responses, rather this is an open question.

In zebrafish, the light–dark cycle does have a large impact on immunity, similar to mammals. Larvae infected with *Salmonella* Typhimurium during the light phase of a light–dark cycle or exposed to constant light displayed enhanced survival, better bacterial clearance, increased neutrophil and macrophage recruitment to sites of infection and enhanced expression of TNFα, IL-8 and IFN-γ compared to larvae infected during the dark phase of the light–dark cycle or those raised in constant darkness [18]. While it is as yet unclear whether this light-dependent regulation of the immune response is dependent on the molecular clock machinery or indeed whether light-regulated neuro-endocrine interactions impose circadian rhythm control of immune responses in fish, there is some evidence for bidirectional immune and nervous system control of circadian rhythms. A recent chemical screen for small molecule modulators of circadian oscillations in fish identified a number of small molecules associated with the nervous system that resulted in phase advances [19] while anti-inflammatory drugs (e.g., NSAIDs, glucocorticoids, cyclosporin) increased circadian amplitudes and small molecule induced inflammation had the opposite effect, suggesting that the immune system feeds back to alter the circadian clock in zebrafish.

### Control of the nervous system by microbiota

Disturbing the microbiota may cause behavioral alteration and even contribute to neurological disorders (Fig. 1), according to the “gut–brain axis” paradigm [20], a concept that has gained popularity and raised hopes that diet and/or probiotics may help stem the rising incidence of neurodegenerative diseases. The zebrafish is a powerful system to test such issues, at least in early development, because of the ease and limited costs of generating axenic (also called germ-free) larvae, which can be recolonized in a controlled way [21]. These axenic or gnotobiotic larvae can then be fed with sterile chow or axenic live foods to be grown for several weeks to juvenile stage, or months to adulthood, although this becomes labor-intensive [22, 23]. Thus, most behavioral assays on gnotobiotic zebrafish are conducted at the larval stage. Initial behavior of larvae, including hunting, sleeping, learning and navigating, can be measured in high-throughput assays as early as 6 days post-fertilization (dpf) [24]. Social interactions (e.g., shoaling behavior) develop in the second week [25]. However, behavioral changes may also be assessed in conventional adults after perturbation of the flora; for example, administration of a probiotic *Lactobacillus* strain induced subtle changes in shoaling behavior and a preference of probiotic-treated fish for a higher position in the water column [26]. This was associated with small but significant changes in microbiota composition, and, more strikingly, to an increase of *bdnf* expression in the brain, as observed in rodents. BDNF (brain-derived neurotrophic factor) is a growth factor involved in various mood disorders [27].

An early study of the behaviors of axenic larvae found an increased spontaneous locomotion compared with conventionally raised or ex-germ-free conventionalized larvae at 6 dpf [28]; this “anxiety-related” behavior was associated with a lower induction of stress-related genes upon osmotic shock, although no causality link was established. The following year, Tal and colleagues reported that locomotion of axenic or antibiotic-treated larvae was higher than that of conventional larvae at 10 dpf, but not 6 dpf [29]. The timing discrepancy with the earlier work was likely due to a lower temperature (and thus a slower development) and/or different lighting programs. A complex microbiota was not needed, as recolonization by single bacterial species prevented this hyperactive phenotype; however, heat-killed bacteria, or typical bacterial molecules triggering innate immune signaling, had no such effect [29]. This requirement for live bacteria suggested involvement of some metabolite. In a follow-up study, the same team showed that the microbiota alter the concentration of 17ß-estradiol, which has a direct impact on the locomotor activity of larvae [30].

Bacteria may also influence the enteric nervous system to their own advantage. In a mono- or duo-colonized zebrafish model, a bacterial pathogen (*Vibrio cholerae*) was shown to displace a harmless commensal species (*Aeromonas veronii*), not only via direct bacterial interactions, but also by impacting gut peristalsis [31, 32]. Live imaging is a major tool in the study of zebrafish host–pathogen interactions, but it requires transparent larvae to be immobilized, which is typically achieved by a combination of anesthesia and embedding in a gel such as low-melting agarose. However, common anesthetics such as tricaine methanesulfonate do not typically block peristaltic movements, making live imaging of the gut extremely challenging. Direct imaging of bacteria in the gut lumen of zebrafish had been reported previously, but usually limited to short time periods [33]. Here, rapid imaging of entire larvae (using light-sheet microscopy, fast enough to take the entire image between two peristaltic events) was performed every 20 min for more than 12 h. This revealed that in fish challenged with *Vibrio cholerae*, gut contractions were not more frequent but were much stronger, resulting in expulsion of *Aeromonas* [31]. Remarkably, this was dependent on the type VI secretion system (T6SS) of *Vibrio*, as was direct killing of *Aeromonas* by *Vibrio*: however, the two events could be dissociated genetically, and loss of modulation of host peristalsis only resulting in an inability of the pathogen to displace the commensal [32].

Microbiota colonize other mucosae than the intestine. The team of Irene Salinas studied the impact of microbiota colonization on the olfactory mucosa [34]. Experiments involving delicate microdissections revealed transcriptional differences of olfactory epithelia between conventional and GF larvae as early as 6dpf. Remarkably, in both larval zebrafish and adult mice, germ-free status resulted in lowered expression of many olfactory receptor genes. This was associated with subtle morphological differences of olfactory sensory epithelia, and suggested a role for the transcription factor NRSF/REST in microbiota-dependent gene repression in the olfactory organ.

At 4 dpf, when the gut becomes colonized, most organs are patent but actively growing and maturing, and the CNS is no exception. By generating germ-free zebrafish larvae and delaying the time of microbial colonization by only three days, Bruckner et al. observed a strong difference in social interactions, even though this behavior becomes apparent only one week later [35]. Thus, they identified a sharp time window during which the presence of microbiota impacts future behavior. By focusing on a population of neurons expressing *lhx8a*, and known to be required for normal behavior, they showed that in colonization-delayed fish, these neurons are in normal numbers, but a subset exhibit exuberant arborescence. Finally, they show that microglial invasion of the forebrain, even though it starts before 4 dpf, is reduced in larvae with delayed microbiota colonization [35]. Therefore, they propose a plausible mechanism by which microbiota promote microglial infiltration of the brain, ensuring proper pruning of dendritic arbors involved in social interactions. This is a remarkable example of the complex interactions that may link microbiota, immune cells and nervous system.

### Control of the immune system by microbiota

The animal immune system has evolved to sense diverse microbial stimuli and relay that information to components of the immune system. As commensal microbiota have been a constant source of stimulation and potential infection to animals during their evolutionary history, the immune systems of zebrafish and other animals do respond to microbiota as a part of their normal life cycle (Fig. 1) [36]. Initial insights into zebrafish immune responses to microbiota were provided by comparison of zebrafish reared germ-free to those colonized with a conventional zebrafish microbiota. That work revealed that microbiota colonization stimulates expression of complement and acute phase proteins [37, 38], and epithelial barrier function [39, 40]. Among professional immune cells, the neutrophil lineage has received the most attention in host–microbiota research in the zebrafish due largely to the relatively early discovery of histochemical [41, 42] and transgenic methods [43, 44] to visualize them. Analysis of gnotobiotic zebrafish revealed microbiota stimulate expression of neutrophil markers [37, 38], neutrophil recruitment to the intestine [39], recruitment to peripheral wounds, and other systemic behaviors [45, 46]. Similar effects on other immune cell lineages are expected, but remain understudied.

Host immune responses to microbiota represent an aggregate reaction to complex stimuli emerging from those microbial communities. However, studies using germ-free zebrafish have shown that these host immune responses are capable of remarkable specificity to individual members of the microbiota or their products [37–39, 47]. Moreover, a subset of host innate immune responses to their own normal zebrafish microbiota can also be evoked by microbiota from the mouse intestine [38], suggesting some common signals emerging from different microbial communities or the ability of host responses to integrate diverse microbial stimuli into a common set of host responses. Several studies have underscored how the host integrates input from complex microbial communities into appropriate immune responses remains an important frontier of research. Early insights were provided by experiments in which germ-free zebrafish were colonized with known mixtures of commensal bacteria, revealing that the impact of a given strain on neutrophil recruitment to the intestine was not necessarily proportionate to the abundance of that strain in the community [48].

Identification of the specific mechanisms by which commensal microbiota regulate aspects of host innate immunity is another important area of research. One major theme is the ability of microbiota to influence host sensitivity to inflammatory stimuli. For example, microbiota colonization leads to increased host expression of intestinal alkaline phosphatase in the intestinal epithelium [39], which in turn detoxifies bacterial lipopolysaccharide present in the gut lumen thereby preventing excessive neutrophil recruitment to the intestine [40]. Another emerging theme is the ability of microbiota to regulate host tissue-specific production of immunoregulatory cytokines. For example, the acute phase protein serum amyloid A (Saa) is induced upon microbiota colonization in the intestine and liver of zebrafish and mammals [38, 47, 49, 50]. In vivo consequences of this microbial induction of Saa have been difficult to resolve in mammals, however, due to complex paralogy and other limitations. Genetic studies in zebrafish revealed that its single Saa homolog serves as a systemic signal to neutrophils to restrict activation, decrease inflammatory tone and bacteriocidal activity while simultaneously enhancing their ability to migrate to sites of peripheral injury [45, 46]. These results underscore that gut microbial colonization can affect distinct aspects of intestinal as well as extra-intestinal immunity.

It is also important to note that the impacts of microbiota on host immunity are not limited to overt influences on the immune system. The presence and composition of microbiota are known to be a major determinant of susceptibility to intestinal pathogens in zebrafish as well as other animals. For example, the ability of *Edwardsiella ictaluri*, the causative agent of catfish enteric septicemia, to cause disease in zebrafish was curtailed by precolonization with an identified set of probiotic bacteria. However, this was not linked with apparent alteration in the inflammatory response to *E. ictaluri* but instead linked to the presence of adhesion factors on the probiotic strains, suggesting physical exclusion of pathogens as a potential mechanism [22]. Similarly, the presence of a commensal microbiota or a define set of commensal bacterial strains protects zebrafish from the fish pathogen *Flavobacterium columnare*, and these effects appear to be independent of *Myd88*-dependent innate immunity [51].

### Control of microbiota by the immune system

As jawed vertebrates, zebrafish possess an adaptive immune system of B and T lymphocytes bearing antigen receptors generated by a semi-random recombination mechanism involving the RAG proteins. Indeed, *rag1*-deficient zebrafish [52] lack both T and B lymphocytes and have become a major tool to study the immune system of zebrafish. In zebrafish, the adaptive immune functions develop a few weeks after initial colonization of the gut by microbiota [53]; accordingly, it has been found that the microbiota composition of *rag1*-deficient and wild-type zebrafish was indistinguishable at 1 week of age, but became significantly distinct from 5 weeks onwards. Strikingly, the abundance of *Vibrio* species, which include major pathogens in fish as well as in mammals, was strongly controlled by a *rag1*-dependent mechanism, with a 4- to 5-log decrease in wild-type adults compared to 1-week larvae or to *rag1* mutants. Transfer of *lck*:GFP + cells, mostly T lymphocytes, to *rag1*-deficient fish, resulted in a rapid decrease of *Vibrio* abundance in the gut [54]. This suggests the presence in these fish of a population of gut-homing *Vibrio*-specific T cells that downregulate the number of these potential pathogenic bacteria.

It should be noted, however, that other groups have reported higher numbers of *Vibrio* in the microbiota of adult wild-type zebrafish [37, 55], highlighting the contribution of other factors such as diet and water properties in final microbiota composition. This has been particularly well illustrated in a 2017 study [56] which also compared the microbiota of *rag1*-deficient and wild-type adult zebrafish. This study found that, while the housing conditions contributed significantly to the final microbiota composition, *rag1* genotype did not. Importantly, wild-type fish tended to be more individually dissimilar from each other than were *rag1* mutants, suggesting that indeed the adaptive immune system does shape the microbiota composition, but in a poorly predictable way. This could be linked to the past history of gut colonization of each individual zebrafish gut which has a stochastic component, even in co-housed animals. The collective *Vibrio*-specific effect observed in the Brugman et al. study (in which *rag1*-deficient and wild-type fish were appropriately co-housed) may be linked to a sporadic event of immunization of these animals; as noted in later studies, this result has yet to be replicated. Additional immunodeficient zebrafish strains have been generated [57], presenting interesting opportunities to test the requirement of other immune system components in host–microbe interactions.

While the contribution of adaptive immunity to microbiota composition was studied in *rag1* mutants, the impact of the innate immune system has been addressed less systematically. This is largely due to the very modular nature of the innate immune system with its many relatively independent components, compared to the integrated properties of adaptive immunity; thus, it is impossible to inactivate the entire innate immune system with a single mutation. Nevertheless, Myd88 is a major adaptor for signal transduction by TLR (toll-like receptors) and IL-1 family receptors, and *Myd88*-deficient animals are commonly viewed as strongly impaired in their innate immune responses. *Myd88* mutant zebrafish are indeed strongly immunodeficient [58] and their microbiota has been compared at 3 weeks of age (i.e., shortly before onset on adaptive immunity) with that of wild-type fish [59]. Interestingly, while the microbiome composition of *myd88* mutants was significantly different to that of co-housed wild-type fish, a stronger influence of housing conditions was observed.

Among innate immune cell types, an impact on microbiota composition has been demonstrated for a major subset of intestinal macrophages lacking in *irf8* mutant zebrafish [60].

With the increased availability of various models of zebrafish with specific innate immune deficiencies, and the democratization of microbiome profiling methods, one may expect a large amount of data in the future documenting the influence of various immune pathways and cell types on microbiota composition. However, the major role of the environment is expected to add a strong source of variability to these assays. It is now clear that such studies require the comparison of co-housed animals, ideally siblings to minimize effects of genetic variation and maternal contributions to the microbiota. It is possible that any difference observed in a given facility will not be replicated in a different one, unless an explicit standardization effort is made by the zebrafish community to make baseline microbiome composition more similar across institutes and facilities.

### Control of microbiota by the nervous system

Like the immune system, the nervous system has primary roles in sensing specific stimuli in the external environment and within the animal’s body, and in mounting appropriate direct and emergent responses. The basal activities and induced responses of the nervous system exert influence over every organ system as well as emergent phenomena such as behavior. It can therefore be expected that diverse aspects of nervous system function directly or indirectly influence the composition and activity of the microbiota. However, due in part to this complexity, it has been relatively difficult to empirically link specific aspects of nervous system function to microbiota composition or activity. To date, the best evidence for nervous system control of the microbiota comes from analysis of the enteric nervous system (ENS) which regulates gut motility and peristalsis (Fig. 1). Zebrafish larvae lacking an ENS due to mutation of the *sox10* transcription factor were found to have elevated intestinal inflammation. This was linked to alterations in gut microbiota composition in *sox10* mutants, including relative enrichment of pro-inflammatory *Vibrio* bacteria. Transplant of microbiota from *sox10* mutant into WT GF recipients led to increased intestinal inflammation compared to GF recipients of a WT microbiota, indicating that the ENS normally functions to limit the proinflammatory potential of the microbiota [61]. This finding was extended using a model in which GF zebrafish were colonized with a simplified model community consisting of a pro-inflammatory *Vibrio* species and/or a commensal *Aeromonas* species, already mentioned above. Though *Aeromonas* was able to persistently colonize the intestines of WT GF zebrafish, introduction of *Vibrio* led to stochastic collapse of the *Aeromonas* population. This was found to be linked to *Vibrio*’s ability to induce intestinal motility, which selectively reduced the *Aeromonas* population without comparable effects on *Vibrio*. The requirement for the ENS and gut motility was demonstrated by showing loss of ENS through mutation of the *ret* tyrosine kinase allowed *Aeromonas* to persist in the presence of *Vibrio* [31]. Similarly, a recent study showed that the fish pathogen *Edwardsiella tarda* also potently induced intestinal motility. Discussed in greater detail in the next section below, the induced increases in peristalsis were required to clear *E. tarda* from the intestinal lumen, without overt effects on intestinal microbial community density [62]. Together these results show that the ENS plays a major role in gut microbial ecology, and that different microbes display differential capacity to induce intestinal motility and differential sensitivity to increases in intestinal motility. As this field continues to expand, we expect that additional aspects of nervous system function will be found to also shape microbiota composition and microbial pathogenesis.

## Sensing of pathogenic infections beyond immune cells

The vast majority of interactions an animal has with its microbial world are with nonpathogenic members of its microbiota, however interactions with pathogenic microbes and resulting infections also occur. Whereas commensal microbial cells are largely constrained to mucosal and skin surfaces, many pathogens deploy specialized mechanisms that allow them to breach those physical barriers to gain access to other host tissues through local and systemic infections. Animals have therefore evolved diverse mechanisms to sense and respond to microbial pathogens at multiple stages of infection. These include primary sensory epithelial cells lining mucosal tissues, detection by various leukocyte lineages, engulfment by and communications with intracellular organelles in various host cells, and detection by organ systems specialized for systemic detection like the nervous system and liver. The role of leukocytes in sensing and responding to pathogens and infections has been reviewed extensively elsewhere [63–65]. Here, we will discuss modes of host sensing of infections and pathogens beyond professional immune cells, with an emphasis on the nervous system.

Studies in mammals have already uncovered important roles for the nervous system as a primary sensor of microbial pathogens. For example, nociceptor sensory neurons directly detect pathogen-associated molecular patterns (PAMPs) and toxins to cause pain. Further, neurotransmitters released by those neurons can feedback onto the immune system to regulate inflammation and infection outcome [66].

### Pathogen sensing in the olfactory organ

Most fish rely heavily on their olfactory sense. Their olfactory organ, exposed to the external environment and thus a potential portal for infection, is associated with a specialized lymphoid tissue [67]. Remarkably, fish are able to detect the presence of some viruses in their olfactory organ. Delivery of medium containing a neurotropic rhabdovirus in the nares of a rainbow trout induces a stronger olfactory response than virus-free medium, which could be attributed to Trka-dependent firing of crypt neurons. This was associated with chemokine mRNA induction within minutes, and later, with a CD8 T cell infiltration in the olfactory organ. In a zebrafish larval model, crypt neuron ablation increased the adult fish susceptibility to a rhabdovirus following bath exposure [68]. The molecular mechanisms involved in this phenomenon remain to be unraveled.

Avoidance or foods or environments with aversive odors is a well-known behavior that diminishes the risk of getting infected. In fish, the best studied evasion behavior is related to predation: a strong alarm response elicited by the smell of a substance release by injured conspecifics, named “Schreckstoff” (“fright material”) by Karl von Frisch almost a century ago. Accordingly, adult zebrafish strongly react to zebrafish skin extract, and fractionation studies have identified the fear-inducing component as a mixture including chondroitin [69]. Directly relevant to the topic of this review, the same group later identified a bacterial component in Schreckstoff [70], implying a role for skin commensal bacteria and suggesting related mechanisms may be used to evade some bacterial pathogens. They also observed a response to Schreckstoff in 5–7 dpf larvae, allowing them to directly visualize the elicited neuronal activity by calcium reporter imaging, and identify the neural circuitry involved in this response [71]. Several groups have now developed medium-throughput setups to analyze the behavioral and neural responses of zebrafish to an array of odorants (e.g., [72]) and rapid progress is expected in this field.

### Pathogen sensing inside the gut

The intestine is a major interface for communications with pathogenic and commensal microbes residing within the intestinal lumen [73] and also with the central nervous system [74]. While professional immune cells serve important roles in responding to microbial encounters in the digestive tract, the epithelial cell layer lining the intestine also serves as key primary sensors of microbial signals which they communicate to the brain and other parts of the nervous system. Among the different intestinal epithelial cell types that may participate in gut–brain communication, particular attention has been given to enteroendocrine cells (EECs). EECs have conserved sensory functions in insects, fishes, and mammals where they are activated by diverse luminal stimuli to secrete hormones and neurotransmitters [75–77]. EECs can form synaptic connections with sensory neurons [78, 79] thereby forming a direct route for gut–brain sensory transmission. Classically known for their ability to sense nutrients [80], EECs in mammals have also been shown to sense microbial metabolites like short chain fatty acids and branched chain fatty acids through G-protein coupled receptors [79, 81]. EECs are also able to sense indole, a microbial catabolite of the amino acid tryptophan [82], but the EEC receptor and potential impacts on the nervous system remained unknown. The intestine is innervated by the ENS as well as sensory nerve fibers from the nodose vagal ganglia and dorsal root ganglia in the spinal cord [83]. Previous work in mice showed that stimulation by the microbial branched chain fatty acid isovalerate can stimulate EECs to activate spinal sensory nerves by releasing serotonin [79]. Whether and how gut microbial stimuli modulate ENS or vagal sensory activity through EECs was still unknown.

Using in vivo real-time measurements of EEC and nervous system activity in zebrafish, Ye and colleagues discovered that the bacterial pathogen *Edwardsiella tarda* specifically activates a subset of EECs through the receptor transient receptor potential ankyrin A1 (Trpa1) [62]. Trpa1 is a highly conserved excitatory non-selective cation channel that can be activated by diverse chemical irritants and has important roles in pain sensation and neurologic inflammation [84]. Microbial, optochemical, or optogenetic activation of Trpa1 + EECs in zebrafish activated vagal sensory ganglia and increased intestinal motility through direct signaling to enteric motor neurons via serotonin secretion. These effects were found to be mediated by tryptophan catabolites indole and indole-3-carboxyaldhyde that are secreted abundantly by *E. tarda* and at lower levels by other commensal gut microbes. These results were also translated to mammals, where these bacterial tryptophan catabolites were shown to also be agonists of human and mouse TRPA1 homologs, and sufficient to induce TRPA1-mediated serotonin secretion in human and mouse small intestine [62]. These results demonstrated that the zebrafish can be used for studying gut–brain communication via the vagus pathway, and uncovered a molecular pathway by which EECs regulate enteric and vagal neuronal activity in response to specific microbial signals in the gut.

## Interactions that result in pathology

### Bacterial infections that result in neuropathology

**Fig. 2:**
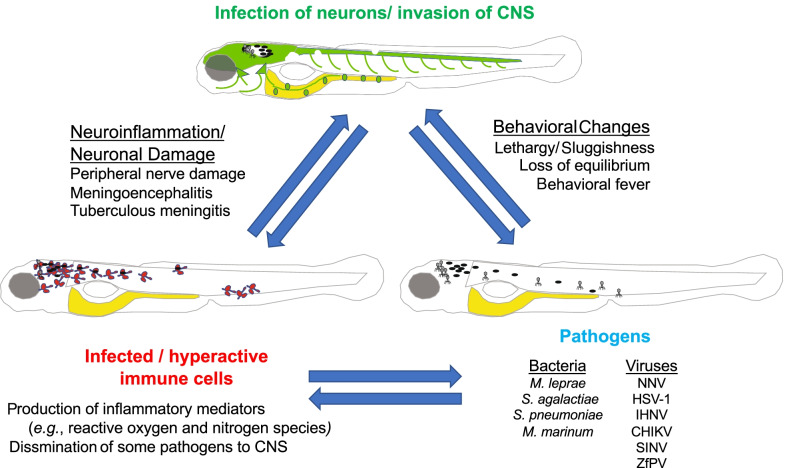
Examples of pathological interactions involving CNS invasion. Pathogens (including but not limited to those listed) may infect immune cells or directly invade neurons or the CNS in zebrafish. Infected immune cells in the periphery may either elicit peripheral nerve damage or cross the blood–brain barrier and invade the CNS, causing neuroinflammation and subsequent neuronal damage. Infection can result in behavioral changes with some similarities to mammals

Perhaps one of the best known examples of an infection-triggered neuro-immune interaction that results in pathology is the peripheral neuropathy following infection with *Mycobacterium leprae*, the bacterium that causes leprosy (Hansen’s disease). Prior in vitro work had suggested that peripheral nerve damage is a direct result of *M. leprae* demyelinating and infecting Schwann cells via an interaction requiring the *M. leprae* outer membrane lipid, phenolic glycolipid 1 (PGL-1) [85]. However, recent work using zebrafish embryos as a model host for *M. leprae* infection has altered this view. Taking advantage of the optical transparency of zebrafish embryos, the study authors directly observed bacterial, glial, neural and immune cell interactions following *M. leprae* infection and showed that while bacteria injected into the dorsal spinal cord were able to alter the myelin structure of glial cells surrounding axons, the bacteria themselves were not observed to directly infect glial cells. Rather, all bacteria were confined to macrophages that themselves directly interacted with and patrolled nerve cell axons [86]. Mechanistically, *M. leprae* PGL-1 stimulated macrophages to produce reactive nitrogen species, which was associated with subsequent mitochondrial and axonal damage in both myelinated and non-myelinated axons (Fig. 2). This work altered the understanding of early events underlying the peripheral neuropathy following *M. leprae* infection. The use of zebrafish embryos to model these interactions is an important advance given the lack of genetic tools in *M. leprae* itself, as it is an obligate intracellular pathogen, and the paucity of genetically tractable hosts to model *M. leprae* infection.

While *M. leprae* infection is associated with damage to the peripheral nervous system, a small number of other bacteria are able to invade the CNS, resulting in meningitis (Fig. 2), including *Streptococcus* species such as *S. agalactiae* (Group B Streptococcus, GBS) and *S. pneumoniae*. In both zebrafish adults and embryos/ larvae, GBS and *S. pneumoniae* can cross the blood brain barrier (BBB) when inoculated intravenously and disseminate into the subarachnoid space and brain tissue [87–89], causing meningoencephalitis that is dependent upon virulence factors (e.g., bacterial toxins, the bacterial capsule and surface anchored lipoteichoic acid) also required in mammalian infection models.

In addition to streptococci, *Mycobacteria tuberculosis* (Mtb), a relative of *M. leprae* that is the causative agent of tuberculosis, can also invade the CNS causing tuberculous meningitis (TBM) following lung infection. While progression to TBM is rare (~ 1%) [90], the mortality rate is high, particularly among patients co-infected with HIV-1 where the mortality rate approaches 50%, while among patients that survive, neurological complications are common. Both adult zebrafish and embryos have been used to model TBM using the Mtb surrogate and natural fish pathogen *Mycobacterium marinum* [91]. Infection of zebrafish with *M. marinum* has been shown to recapitulate a number of features of human Mtb infection, including granuloma formation [92]. Intraperitoneal infection of adult fish with *M. marinum* results in the formation of Rich foci, granulomas localized to regions of the brain, spinal cord or meninges, in ~ 20% of infected fish [91]. In embryos and larvae, however, the rate at which *M. marinum* can invade the CNS is correlated with formation of the BBB. In zebrafish, the BBB begins to be formed at 3 dpf, when the tight junctions between endothelial cells that separate the blood and brain tissue form. Size dependent exclusion of small molecules and proteins begins to be observed at 3 dpf in some but not all cerebral vessels and gradually matures over the next 7 days [93]. Embryos intravenously infected with *M. marinum* prior to BBB formation (2 dpf) display bacterial dissemination into the brain as early as 1 day post infection while infection of larvae at 4 dpf, after tight junctions have been formed, show a delay in the ability of *M. marinum* to invade the brain parenchyma. Interestingly, *M. marinum* appears to cross the BBB via a ‘Trojan horse’ mechanism, where infected macrophages cross the BBB and establish granulomas within the CNS. Alternatively, in the absence of macrophages, *M. marinum* can pass the BBB via transcellular migration (*i.e.*, invasion of brain endothelial cells) in a process that is dependent on *M. marinum*’s ESX-1 type VII secretion system [94].

Importantly, observations made using zebrafish as a model host have been shown to translate to human TBM. A forward genetic screen for host genes required for control of *M. marinum* replication in zebrafish identified the *lta4H* gene [95], whose product catalyzes the production of the pro-inflammatory eicosanoid LTB4. Its overexpression results in a pro-inflammatory state with excessive eicosanoid LTB4 production and increased levels of TNFα expression while it’s deficiency results in an anti-inflammatory state with production of the lipoxin LXA4 and decreased levels of TNFα. Further work identified one SNP in the human homologue of *lta4H* that affected the transcription of the gene, with one allele predicted to result in increased levels of transcription and a pro-inflammatory state and the other allele in lower levels of transcription and an anti-inflammatory state [96]. Interestingly, TBM patients homozygous for the pro-inflammatory *LTA4H* allele showed greatly increased survival following dexamethasone treatment compared to patients homozygous for the anti-inflammatory allele where dexamethasone treatment was more detrimental to survival or compared to the patient population studied as a whole, independent of host genotype, where dexamethasone treatment only had a very modest effect on patient survival [96]. This study is particularly important as it demonstrates how studies in fish do translate to human infection and have the potential to influence therapeutic interventions for conditions like TBM.

### Viral infections that lead to pathology

Infection of the CNS by pathogens will cause behavioral alterations, either by directly affecting neurons or via the indirect effect of neuroinflammation (Fig. 2). Behavioral anomalies, ranging from lethargy to overactivity, are often the first signs suggesting that a fish may be infected [97]. We will here focus on viral infections of the zebrafish CNS and what is known of their impact.

Most of our knowledge on viral infections in zebrafish stems from experimental infection systems with viruses from other fish, and sometimes from humans, as no natural zebrafish virus was known for a long time [98]. This has recently changed, as a picornavirus named ZfPV has been detected in laboratory zebrafish and found to be widespread in facilities worldwide [99]. ZfPV tropism is principally intestinal, but it was detected in the brain of the rare CG2 strain [100]. A similar situation is observed for several human viruses, including major pathogens such as polio virus (also a picornavirus). Pathological or behavioral data have yet to be reported for zfPV infection in CG2 zebrafish.

NNV (nerve necrosis virus), a nodavirus, is the causative agent of viral nervous necrosis or viral encephalopathy and retinopathy in multiple tropical and temperate farmed marine fish species [101]. A zebrafish model of NNV infection has been established, and viral tropism, including highest amounts in the brain, was found to be comparable to that observed in natural hosts. Pathological lesions were observed in infected adult brains by histology, yet other symptoms were not reported. Mortality was very rapid in larvae, associated with a higher viral titer and much weaker type I IFN response [102].

A zebrafish model has been established for Herpes Simplex Virus 1 (HSV-1), a human herpesvirus with a well-known tropism for the CNS and a major cause of neonatal encephalitis in infants. In adult zebrafish, following i.p. injection, HSV-1 replication was modest and transient, but the virus was shown to move from the abdomen to the brain, where it was detected in neurons and persisted longer than in other organs [103]. Mortality occurred when fish were immunosuppressed with cyclophosphamide; behavioral signs were not reported. An ulterior study performed in larvae showed them to be much more susceptible to HSV-1 than adults, and were found to lose their typical brisk locomotor reactivity [104].

Infectious Haemorrhagic Necrosis Virus (IHNV), a rhabdovirus, is an important pathogen in salmonid aquaculture. Salmonids live in cold waters, and the virus normally does not replicate above 18 °C; however, a strain adapted to growth up to 25 °C was found to replicate in and to kill zebrafish larvae maintained at 24 °C [105]. A stereotyped suite of signs was observed before the death of the infected larvae, including loss of equilibrium and then loss of reaction to touch, which was concomitant with invasion of the brain by the virus. However, the vascular endothelium was the primary target of the virus, causing widespread vessel disruptions and hemorrhages. Interestingly however, a transcriptomic analysis of this infection revealed a specific decrease of *epd* transcripts, encoding ependymin, one of the most abundant proteins in CSF. This was linked to the rapid loss of *epd*-expressing cells surrounding the main cerebral vessels [106].

Chikungunya virus (CHIKV) is a re-emerging arbovirus of humans causing muscular and articular signs in adults but also encephalitis in infants. This virus replicates very well in zebrafish larvae, however the strong type I IFN response induced in larvae allow them to control the virus and survive. After IV inoculation, the virus replicates first in the periphery, then propagates to the CNS, where it persists much longer; this is probably linked to the relative lack of IFN response in the brain [107]. Sindbis virus (SINV), a virus extensively used to model viral encephalitis in mice, was also found to replicate in zebrafish larvae. SINV and CHIKV are closely related and the two infections share many similarities in zebrafish larvae, including progression from periphery to CNS [108] and strong IFN induction [109]. Surprisingly however, the two viruses preferred different routes to cross the BBB—infecting the endothelial cells for CHIKV, vs axonal transport for SINV [108].

Loss of equilibrium and sluggishness, sometimes transitory, was often observed in CHIKV- or SINV-infected larvae, and was one of the parameters included to score disease severity [107]. Nevertheless, heavy infection of the brain, readily detectable in live animals using fluorescent reporter viruses, did not always correlate with locomotor impairment. This is probably linked to the strong individual variability in infected brain areas [108].

To summarize, a variety of neuroinvasive viruses can infect zebrafish, and behavioral phenotypes have been observed, even if not documented in great detail. One important question remains to be addressed in these models: what are the relative contributions of infection itself and of the inflammation it causes in these phenotypes? The development of new tools to generate well-controlled sterile neuroinflammation will be required to disentangle these effects.

### Behavioral fever

We are all familiar with one outcome of an interaction between immune and nervous systems: fever. Our body temperature is regulated by the hypothalamus which instructs the sympathetic output system. Its set point is upregulated by plasmatic pyrogens, most notably pro-inflammatory cytokines such as IL-1 and IL-6, which access specific CNS areas through the permeable capillaries of the circumventricular organs. These cytokines trigger microglia to release prostaglandin E2 (PGE2), which in turn acts locally on temperature-regulating neurons in the hypothalamus [110].

While ectothermic animals cannot use internal mechanisms to increase their body temperature, sometimes they may use the environment to warm themselves. When presented with a choice in water temperatures, infected fish typically prefer warmer areas than uninfected controls (Fig. 2), a phenomenon identified decades ago and dubbed “behavioral fever” [111]. The existence of behavioral fever has been established relatively recently in adult zebrafish [112]. This article showed that when allowed to move in a thermal gradient, compared to fish constantly kept at their normally preferred temperature of 28 °C, fish challenged IP with polyI:C (a viral PAMP mimic) displayed a stronger antiviral transcriptional response. Furthermore, when provided with this gradient, they withstood a SVCV (spring viremia of carp virus) challenge with no disease signs, unlike fish kept at 28 °C. The latter effect was probably linked to the temperature sensitivity of the virus itself besides the heightened immune response. A few years later, the same group tested if behavioral fever was detectable in zebrafish larvae [113], and identified its onset at 18–20 dpf. This suggests that the coupling of the immune and nervous system to induce thermal response occurs late in development. However, this was tested using polyI:C immersion, which is, in our experience, less efficient to trigger an immune response than IP injection, and much less than many infections. It thus remains possible that stronger stimuli and/or real infections may trigger behavioral fever at earlier stages, but this remains to be tested.

## Conclusions

The literature reviewed here underscores the broad utility of the zebrafish as a model for studying the complex and reciprocal interactions between the nervous system, the immune system, and commensal and pathogenic microbes. Most of these insights have been afforded by the capacity of the zebrafish system, which continues to be a distinct advantage of this non-mammalian vertebrate model. Also fueling these discoveries are the rapidly emerging genetic tools and resources in the zebrafish for these organ systems. Future expansion of the field will continue to require more tools for imaging and manipulating specific neural and immune cell types and subtypes, as well as their signaling and other activities. Also, the tools available for visualizing and genetically manipulating most of the microbes reviewed here are quite limited, and further resource development is warranted. We also anticipate that increased application of genetic screens in both host and microbe as well as chemical and toxicological screens in the zebrafish system will increase the value of this non-mammalian system to the broader field.

Although these zebrafish studies have provided insight into neural-immune–microbial interactions, most of these available studies have focused on pairwise neural–immune, immune–microbial, microbial–neural interactions. By contrast, and perhaps not surprisingly considering the added complexity involved, relatively few studies have considered all three components together. There is therefore a need for future studies that consider together the immune and nervous system as well as microbial interactions, in order to more rapidly advance the field. One can predict that such studies would provide powerful insights into the mechanisms of complex diseases known to involve these three components, such as inflammatory bowel disease, psoriasis, obesity-related inflammation, neonatal infectious encephalitis, psychiatric diseases associated with microbial dysbiosis during early life, to cite only a few that can be modeled in zebrafish.

The majority of the discoveries described above have also focused on individual life stages with the vast majority during relatively early embryonic and larval stages. Considering that the immune and nervous systems undergo continual maturation and education throughout the lifecycle, it will be interesting to determine how these reciprocal interactions manifest at different life stages. Further, the zebrafish is well positioned to evaluate the impact of prior immune and neural experience on later stage interactions and phenotypes. For all these reasons, we anticipate that the zebrafish model will continue to serve as a valuable non-mammalian model system in future studies in this field.

## Data Availability

Not applicable.

## Abbreviations

BBB: Blood–brain barrier
BDNF: Brain-derived neurotrophic factor
CGRP: Calcitonin gene related peptide
CHIKV: Chikungunya virus
CNS: Central nervous system
CSF: Cerebrospinal fluid
Dpf: Days post-fertilization
EEC: Enteroendocrine cell
ENS: Enteric nervous system
GABA: Gamma-aminobutyric acid
GBS: Group B Streptococcus
GF: Germ-free
HPA/I: Hypothalamic–pituitary–adrenal/interrenal
Hpf: Hours post-fertilization
HSC: Hematopoietic stem cell
HSV: Herpes Simplex Virus
IFN: Interferon
IHNV: Infectious Haemorrhagic Necrosis Virus
IL: Interleukin
IRF: Interferon-responsive factor
JAK: Janus-associated kinase
KAR: Kainate receptor
MAMP: Microbe-associated molecular pattern
MTb: *Mycobacterium tuberculosis*
NNV: Nerve Necrosis Virus
NRSF/REST: RE1 silencing transcription factor or neuron restrictive silencer factor
NSAID: Non-steroidal anti-inflammatory drug
PAMP: Pathogen-associated molecular pattern
PGE2: Prostaglandin E2
PGL: Phenolic glycolipid
RAG: Recombination activating gene
SAA: Serum amyloid A
SCN: Suprachiasmatic nuclei
SINV: Sindbis Virus
STAT: Signal transducer and activator of transcription
SVCV: Spring viremia of carp virus
TBM: Tuberculosis meningitis
TLR: Toll-like receptor
TNF: Tumor necrosis factor
Treg: Regulatory T cell
TRPA1: Transient receptor potential subfamily A1
WT: Wild-type
ZfPV: Zebrafish picornavirus
